# Elementium: design and pilot evaluation of a serious game for familiarizing players with basic chemistry

**DOI:** 10.1007/s10639-023-11791-9

**Published:** 2023-04-18

**Authors:** Alexandros Filippas, Stelios Xinogalos

**Affiliations:** 1 Graduate Program of the Department of Applied Informatics, University of Macedonia, Thessaloniki, Greece; 2 Department of Applied Informatics, University of Macedonia, Thessaloniki, Greece

**Keywords:** *S*erious games, Chemistry, Game design, Game evaluation, Survey

## Abstract

Serious games (SGs) about Chemistry have the potential to cope with challenges, such as students’ low performance and lack of motivation for the subject. However, the majority of existing SGs for Chemistry have the form of educational applications infused with some elements of entertaining games. The aim of the study presented was to design and evaluate a new SG with rich game mechanics for Chemistry. The game is called Elementium and revolves around basic topics of Chemistry, such as chemical elements and compound terminology, creation and everyday usage of such elements. The main goal of the game is to familiarize junior high school students with the aforementioned subjects. The design of Elementium was carried out implementing the dimensions described in the Four-Dimensional framework, as proposed by de Freitas and Jarvis in 2006. After the development process, Elementium was evaluated by people in the field of education that are currently teaching or have taught Chemistry in the past. The participants play-tested the game at leisure in their homes and evaluated it based on the key criteria for SGs design proposed by Sanchez in 2011, as well as other quality indicators established in the literature. Elementium was positively evaluated by Chemistry teachers in terms of its acceptance, usability, didactic utility, and game environment. The positive results concluded from this evaluation show that Elementium is fulfilling its main purpose and can be used as a supplementary tool in the teaching process. However, its true didactical effectiveness has to be confirmed through a study with high school students.

## Introduction

Serious games (SGs) comprise a dedicated category of video games whose primary objective is not entertainment, rather educating individuals through entertainment (Zyda, 2005). Nowadays, video games are increasingly utilized in such sectors as health and wellness, professional learning, culture and education, with the latter making up for more than half of the SGs in all domains (Calderón & Ruiz, 2015). Educational serious games integrate teaching material into a video game environment, aiming to provide knowledge in the shape of entertainment, in order to support younger students in the comprehension of demanding topics. Many SGs addressing students are used in the ambit of school classes or are created to be used during the player’s free time. Despite their main purpose being the education of the user, SGs must maintain a balanced ratio between the didactic content and the entertainment given, in order to be attractive (Abt, 1970).

According to a study by Connolly et al. (2012) examining the potential impact of SGs on their users, playing computer games aids knowledge acquisition and content understanding and can have positive motivational outcomes. Similar results are recorded by Young et al. (2012). In this study it is found that SGs have ‘very promising impacts’ on school classes and the authors suggest that teachers should incorporate video games in their classrooms. Regarding SGs designed for Chemistry, Hu et al. (2022) carried out an extensive meta-analysis about game-based learning (GBL) and its potential impact on Chemistry learning. The study implies that GBL is a good aid for Chemistry and suggests that certain game design features may help Chemistry education to cope with challenges like low performance and motivation. On the other hand the study concludes that *“more value-added research is needed to identify more effective game design features and instructional design features and provide design guidelines for chemistry GBL”* (Hu et al., 2022; p. 1531).

The aforementioned conclusion by Hu et al. (2022) along with the current state of the art in SGs for Chemistry motivated the work presented in this article. Specifically, our search and comparative analysis of SGs for Chemistry showed that the majority of existing games do not incorporate rich game features, such as missions, enemies, non-playing characters (NPCs), and inventories. We have to note that this result was not surprising, since several researchers have highlighted during the last decade that educational and game design principles have not yet been adequately integrated (Bellotti et al., 2011; De Gloria et al., 2014) and this does not allow us to make the most out of SGs both in learning and entertainment. Although several serious game design frameworks have been devised and are available nowadays, they do not *“distinguish the fun factor from the learning contents well”* (Silva, 2020; p. 1); making difficult the design of games with a balance between education and fun, even when this is a result of cooperation between pedagogy experts and games experts (Silva, 2020). As a matter of fact, Natucci and Borges (2021) consider the achievement of such a balance between pedagogy, emotions and game elements to be one of the greatest challenges for pedagogy and game experts in the next few years.

So, the current work regards designing and implementing a SG with various game features found in role-playing games (RPGs) that are tightly connected with the educational content through the game’s scenario, having as an ultimate goal to familiarize users with the basic concepts and terminology of Chemistry. The game, called Elementium, incorporates material based on the textbooks of Chemistry courses that are currently being taught in junior high schools in Greece. The conceptual understanding in Chemistry contains the ability of someone to think both at a macroscopic and microscopic level. That, in combination with the vast number of chemical elements and compounds that the student has to memorize, can often trouble both the educator and the learner. Our aspiration for developing this SG is to provide supplementary material that will enrich the learning experience and will act as a motivation for students to self-study and improve their knowledge. In this article, a pilot study is carried out in order to investigate teachers’ perceptions regarding the game in terms of its acceptability, usability, didactic utility, and game environment using the key criteria proposed by Sanchez (2011), as well as other quality indicators established in the literature. Consequently, the main research question of this study is:

*What are teachers’ perceptions on the acceptability, usability, didactic utility, game features, and balance between game and learning of the RPG Chemistry game Elementium?*

The rest of the article is structured as follows. Section 2 briefly reviews the results of studies on using educational games in various Chemistry fields. In Sect. 3 the design process and the main characteristics of the educational game Elementium are presented, while Sect. 4 introduces the methodology that we followed for the pilot evaluation of the game. Section 5 presents the results of the study, which are discussed in Sect. 6. Finally, in Sect. 7 the limitations of the study are presented, while in Sect. 8 conclusions are drawn.

## Background

There are several SGs that engage with Chemistry topics, mostly targeting younger kids or beginners. In this section the results of studies utilizing such games are presented. Furthermore, other SGs about Chemistry that were found in the literature are analyzed based on the main axes proposed in the *Four-Dimensional design framework for Serious Games*, as proposed by de Freitas and Jarvis (2006) and revisited by de Freitas et al. (2009).

Shui (2013) presented *Rainbow City*, a ‘computer-aided learning software’ targeting high school students. During gameplay the user can collect chemical equations and necessary ingredients in order to conduct chemical experiments, safely, in the virtual lab contained in the game. The player can navigate through the game world and interact with non-player characters (NPCs) or other online players, asking them questions about the experiments that need to be conducted. These ‘self-generated’ questions are, according to the author, of higher value, when compared to passive listening during traditional teaching.

Fontana (2020) developed a SG called *Molecule Madness*, a gamification of the software ChemDraw, aiming to aid the teaching and social interaction of a class during the COVID-19 pandemic. The game, in the form of a single-elimination tournament, is hosted online with the help of web-conferencing software. The results of this study showed that using *Molecule Madness* as a substitute for the postponed laboratory sessions can improve the wellness of the classroom and have a positive impact on student morale.

Soares et al. (2018) implemented *World of Chemistry*, a virtual reality SG with narrative elements, meant to motivate students to study Chemistry. A study was conducted with the participation of volunteers that played the game and the results showed that the game was a good motivational influence for the majority of them.

Besides the aforementioned SGs and their respective studies, other SGs related to Chemistry topics can be found online, using keywords such as ‘serious games’ and ‘Chemistry’. The games found are analyzed in Table 1 according to the game features they incorporate and the four dimensions of the *Four-Dimensional framework for Serious Games* (de Freitas & Jarvis, 2006; de Freitas et al., 2009), which are the following:

- *Context*. The context of the game refers to elements, such as: the place that the game will be used in, such as a classroom or an outside place; the way of accessing the game; the existence or not of technical support.
- *Learner*. The learner dimension refers to the following elements of the learner or learner group that the game aims at: demographics (age, gender, culture); preferences; skills in Information and Communication Technologies and game experience.
- *Representation*. This dimension deals mainly with: the ways that immersion is achieved; the level of fidelity both in terms of the graphics and the representation of the game world; the interaction of the player with the game. The elements in this dimension of the framework are clearly connected to the game features (presented in a separate column in Table 1) and contribute to the engagement and entertainment of the player.
- *Pedagogy*. This dimension is concerned with the underlying learning theories utilized and gives emphasis to the associative, cognitive and situative perspectives of the learning process.

Through the comparative analysis we conclude that even though there are plenty of SGs that concern Chemistry, the vast majority of them fall short when it comes to the incorporation of rich game features (e.g. missions, enemies, inventories) that are considered not only to entertain, but also to motivate (Connolly et al., 2012; Papastergiou, 2009) and engage players/learners (de Freitas & Jarvis, 2006), as well as enhance their performance (Papastergiou, 2009). Specifically, most of the games comparatively analyzed resemble educational software applications that are meant to teach specific Chemistry topics and only partially adopt video game features. We must note that educational software applications with or without game elements can also enhance learning, but serious games have the potential to deliver more benefits to students, who are widely characterized as digital natives due to their inclination on computers, video games, and the Internet (Prensky, 2001). Having as our main goal to contribute to the field of SGs for learning Chemistry and the need for research on more effective game design features (Hu et al., 2022), we aimed at: designing an RPG SG for Chemistry that incorporates the educational content into a fully functional video game, with its own game mechanics and attractive scenario; and investigating the overall acceptance of the game design and its potential impact on learning Chemistry.

**Table 1:** Comparative analysis of free SGs regarding Chemistry

Title	Game features	Context	Representation	Learner	Pedagogy
ChemRacer 2713: The Legend OF Kid Chem (1999) (no longer available)	genre: educational -20 identical ‘tracks’ -the opponents belong to different teams representing periodic table groups -the player captures opponents and proceeds to answer a question related to their respective group	-single-player -free time -basic guide	-top-down -static levels	-requires little to no prior knowledge -for students	-element terminology and properties -glossary -no feedback at wrong answers
Chemicus: Journey to the Other Side (2001) (no longer available)	genre: educational adventure -the player clicks on the screen in order to navigate the world, collect items and solve puzzles	-single-player -free time -basic guide	-3D static scenes and cutscenes	-requires good knowledge -for students	-organic Chemistry, atomic structure, chemical bonds
US ARMY STARS: ELEMENTS (2022)	genre: interactive − 4 game sections containing a periodic table, an ‘atom builder’ with real-time visualization, a section with various quiz sets and a compound creation based minigame	-single-player -both free time and classroom -basic guide and preferences	-interactive periodic table -3D molecule models	- requires good knowledge -for older students and adults	-periodic table,subatomic structure, stability, reactions -feedback provided for wrong answers
Outer Space Molecule Chase (2022)	genre: adventure -10 unique levels -player avoids enemies, collects atoms and creates compounds to overcome obstacles	-single-player -free time -basic guide in game’s webpage	-top-down -poor quality graphics	- requires little to no prior knowledge -younger students	-chemical compound creation and use for puzzles
Balancing Chemical Equations (2021)	genre: simulation -the player manipulates the two parts of a chemical equation in order to balance it, while visualizing the results	-single-player -both free time and classroom -basic guide and tutorial	-2D representation	- requires good knowledge -for older students and adults	-chemical equation balance
Organic Pop (Keewon, nd)	genre: Puzzle -based on the octet rule the player connects atoms in order to create the given chemical compound	-single-player -both free time and classroom -basic guide and tutorial	-2D representation	- requires good knowledge -for older students and adults	-chemical bonds, compound creation

The genre of the game, namely RPG, was selected taking into account relevant literature. Cheng et al. (2015) in their literature review of empirical research on the use of SGs in science education from 2002 to 2013, concluded that the most popular game genre among SGs was adventure or role-playing games. The same study concludes that the use of avatars in adventure RPGs *“allows students to participate in inquiry activities and to explore different tasks embedded in the game environment repeatedly”* (Cheng et al., 2015; p. 370) and notes that RPGs might more easily provide students with the experience of immersion.

Our choice was further reinforced by studies which show that RPGs have a great impact on personal learning and development skills (Baptista, 2015), and moreover have educational benefits as they promote reading in a recreational way, they are useful for memorizing tasks and allow access to knowledge in a meaningful way (Grande-de-Prado, 2020).

## Design of Elementium

Elementium is an adventure 2D video game with elements and aesthetics of retro RPG games, the principal idea for which came from the game Outer Space Molecule Chase. Alongside the results of the comparative analysis carried out, for the design process of our SG, the Four-Dimensional design framework (de Freitas & Jarvis, 2006; de Freitas et al., 2009) was taken into account.

As for the first dimension, that is the *general context*, Elementium was designed to be played as a single-player PC game. The mean gameplay time is calculated at 1.5 h for the average player, so it is meant to be played during the user’s free time and not during a school class. The user can find the 32-bit or 64-bit installer, along with extra information, at the dedicated webpage that was created.

The second dimension is about the *representation* and the priority at this design step is the immersion of the user. Even though a higher level of engagement is succeeded with more realistic graphics, that is not always the case. As de Freitas and Jarvis (2006; p. 4) underline, *“verisimilitude can distract from learning outcomes and in some cases confuse learners”* and *“not all learners learn well in immersive environment”*. Furthermore, as Cairns et al. (2014) note for digital games in general, gamers do not consider game play to be less important than appearance, while there is an increasing interest for retro-style games that use limited and low resolution palettes to produce video games characterized by a rich player experience. The same study notes that any of the distinct three levels of immersion is considered to be a good feature in a game. Taking those studies into consideration, we attempt to achieve, at least, the first level of immersion, namely the engagement of the player, and we choose retro-style graphics and an appealing scenario with reasonable progression. Additionally, to match the retro theme and to further enhance the sense of a continuous rhythm, a subtle looped soundtrack is used. The player is constantly assisted so that mistakes are avoided, while during the quiz questions, suitable feedback is provided both in correct and incorrect answers.

The third dimension pertains to the *learner*. Elementium contains basic level Chemistry topics, while the educational context was based on the Chemistry textbooks of the 2nd and 3d grade of Greek junior high school. Although, it is addressed mainly to students, due to the very design of Elementium that requires little to no prior Chemistry knowledge, it can be played and be beneficial for anyone interested in the subject.

The final dimension of the Four-Dimensional design framework is about *pedagogy*. The main aspect of this dimension is that through the game, the player must be able to obtain knowledge that will later be useful in a school class or in everyday life. The player is provided with information not only about the terminology of chemical elements and compounds or the various possible combinations of elements, but also information about the common names and uses of many compounds. The cognitive subject, that is the familiarization with the aforementioned concepts of Chemistry, is successfully transmitted to the student through the understanding and creation of the proper chemical compound depending on the situation and the final quizzes of each area.

According to the scenario of Elementium, the user plays the role of a junior high school student who is preparing for an upcoming Chemistry exam but somehow ends up on a strange alien planet, trying to find his way back home. He will have to fight enemies, complete quests and answer sets of Chemistry based questions at the end of each map region in order to finish the game. Elementium is implemented with the help of the Unity game engine and the scripts are written in the programming language C#. There are more than 70 scripts, containing around 265 classes and 5000 lines of code. The following part of this section presents the game flow and the main mechanics of Elementium. In parenthesis, a reference to the corresponding design framework dimension that each gameplay element supports is noted.

**Fig. 1:**
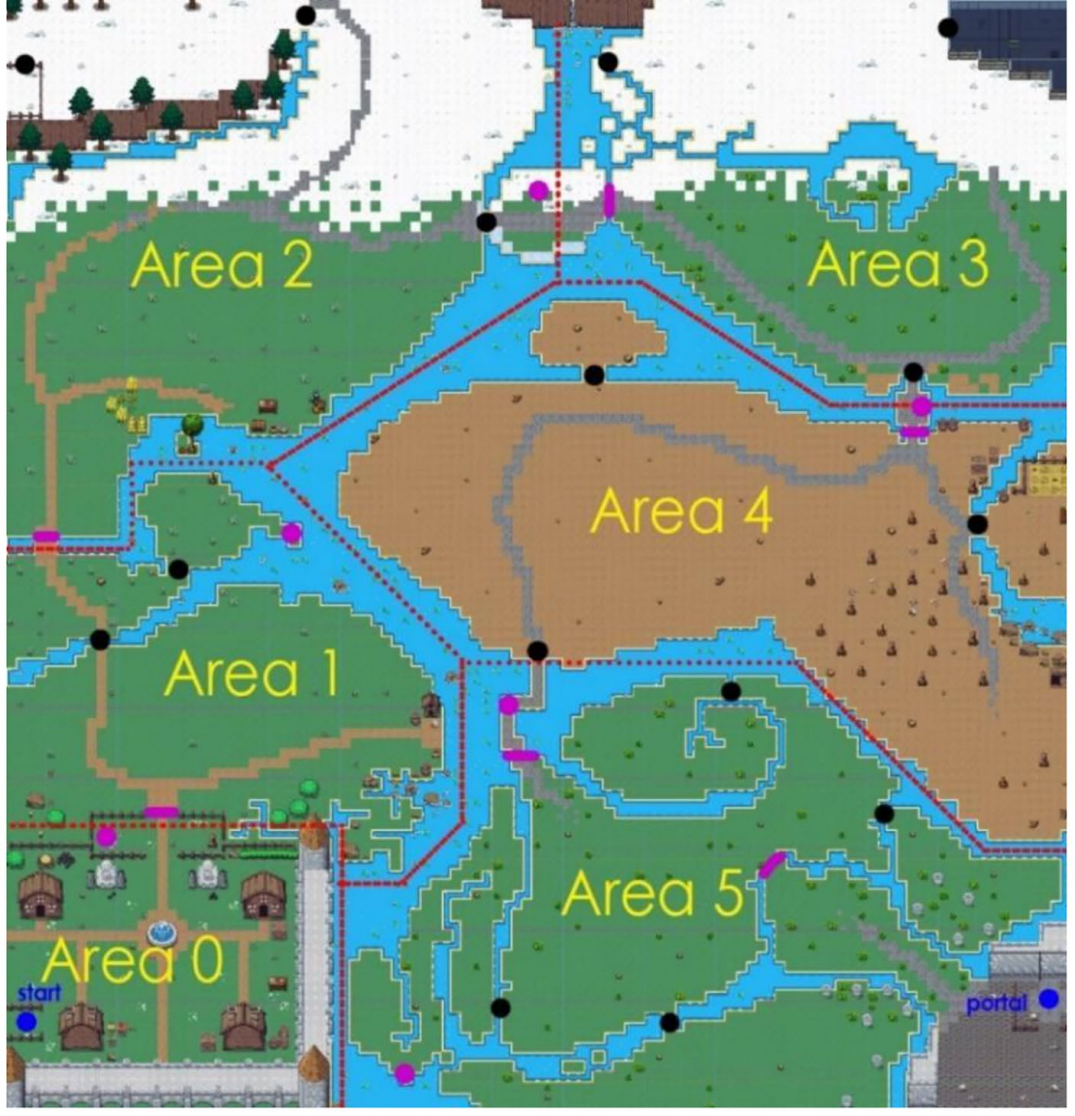
Map of Elementium with main areas marked, along with the starting and ending points and question related obstacles (purple dashes) and their respective NPCs (purple dots). Black dots represent Chemistry related obstacles

The player is initially presented with the main menu, from where some informational content can be viewed, the application can be terminated or the main game can start, by choosing either English or Greek as a language (learner: mother language). The first scene of the game shows the main character studying in his room, while a message prompts the player to interact with the environment around him by clicking the right mouse button (representation: interactivity). After clicking on the open book on the desk, a dialogue sequence starts following the scenario of the game and at the end the character is transported to the main scene. Besides the contribution to the story, the purpose of the first scene is to familiarize a new player (context: support) with the main character’s movement and interaction system and with the dialogue and question windows (representation: interactivity, game world representation). The main scene of the game is set on a uniform map consisting of six areas, labeled 0–5 in Fig. 1.

**Fig. 2:**
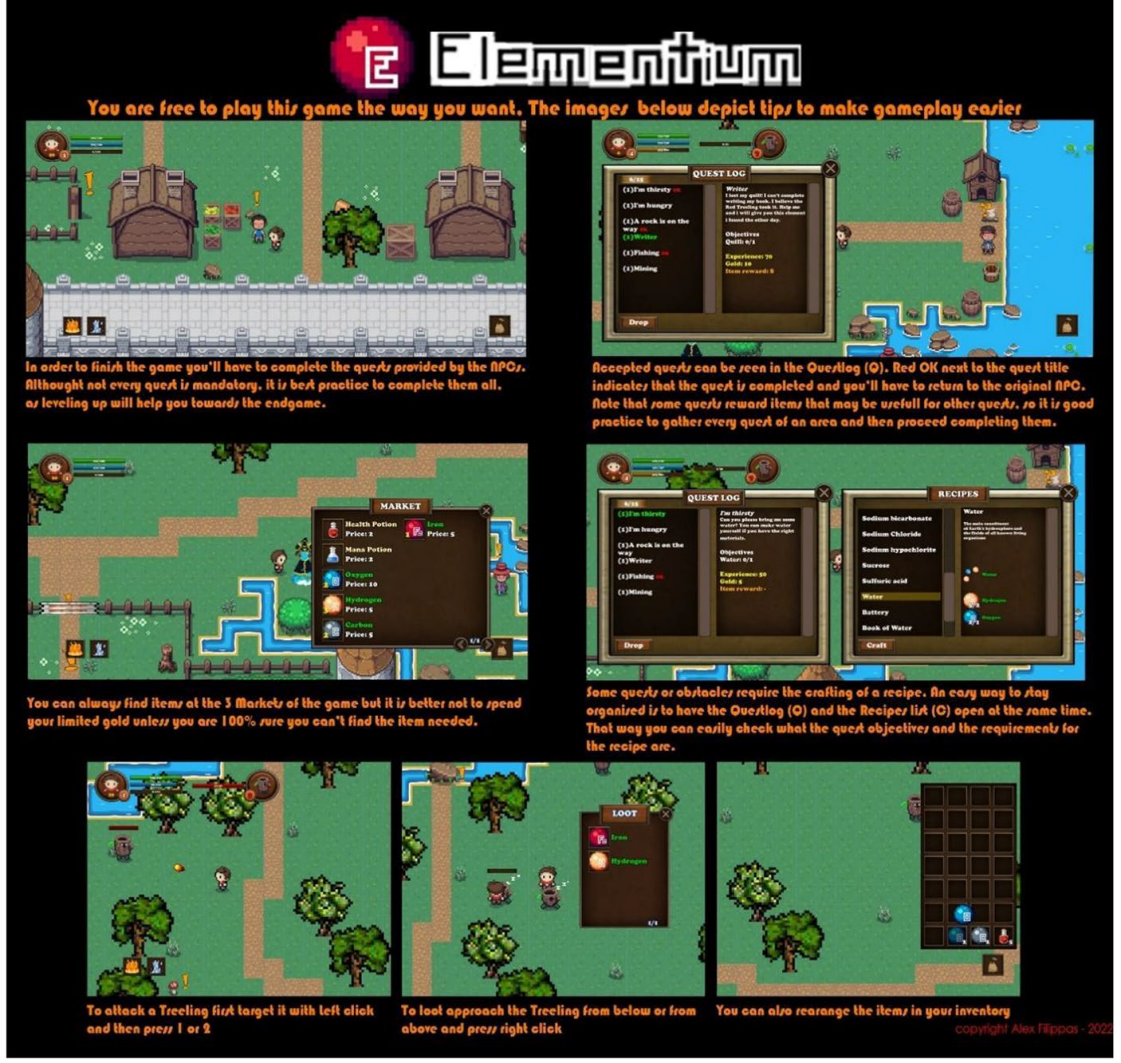
The detailed gameplay tutorial. The player can access this tutorial anytime, throughout the game menu

The first area is enclosed inside the walls of the town of Mendeleev, where the player encounters the first NPC character that explains the basic functionality of the inventory, the crafting system and the help window (context: support). In order to leave the town, the player must find the key to the main entrance which comes as a reward after the three interlocked quests are completed (representation: immersion). The quests are carefully chosen as representatives of the three types of collection quests that the player will face during the game: crafting quests, gathering quests and quests that the player has to find a quest item (pedagogy: associative, cognitive, situative). After the main gate is unlocked, the player encounters another gate and a ‘teacher’ NPC, which holds Chemistry questions (pedagogy: cognitive). If the player answers all of the questions correctly, the door can be unlocked. At all times, the player can press the escape button on the keyboard, to reveal the game menu window (context: support), containing gameplay info. In the game menu there is also a checkbox to mute the in-game music, a button that opens the save menu and a button that opens a webpage containing a more detailed gameplay tutorial (context: support) (Fig. 2).

In the second area the player gets introduced to Treelings, the enemies of the game and obstacles that block the path (representation: immersion, game world). Treelings come in three types, namely normal, ranged and fast, as well as boss variations of each. The obstacles are implemented in the form of NPCs, so it is easier for the user to navigate through the tasks. At the end of this area there is again a teacher NPC with Chemistry based questions (pedagogy: cognitive). The rest of the game follows the same pattern of increasing difficulty Chemistry related quests and questions at the end of each area, with few exceptions of interconnected quests from different areas (representation: immersion). At the end of the final area, the player must face the final enemy that guards the portal to Earth. After activating the portal, the character returns to his room and immediately goes to his classroom for the Chemistry exam. There, the player must answer 15 final questions in the form of an exam, to complete the game (representation: immersion, interactivity).

The main character of the game can move using either the W, A, S, D keys or the keyboard arrows and can execute two ranged attacks (representation: interactivity). The attacks can only be executed if two conditions are met: the player has to select an enemy with a mouse click and the character has to face the enemy. When a spell is casted a horizontal progress bar appears and fills based on the casting time of the spell and an amount of mana points is consumed. The main character starts at level 1 and gains experience points (XP) by completing quests (pedagogy: associative, cognitive) or eliminating enemies (learner: game experience).

By selecting an enemy, a UI element appears at the top of the screen, informing the player about the enemy level (context: support) and real-time health points. Enemies try to deal damage to the main character, if they get attacked or if the character walks in their attack range and in both situations the character enters the combat state. The health points of a character and an enemy replenish when out of combat. If the character’s health points reach zero, he immediately respawns at the starting point, in the town. Respectively, if its health points reach zero, the enemy enters a sleeping state from which it recovers after 2.5 min. When the enemy is in the sleeping state, the player can take its loot, which is crucial for the progression of the story (representation: immersion). Loot includes elements, potions and quest items and it is only available the first time the enemy is eliminated (learner: skills).

**Fig. 3:**
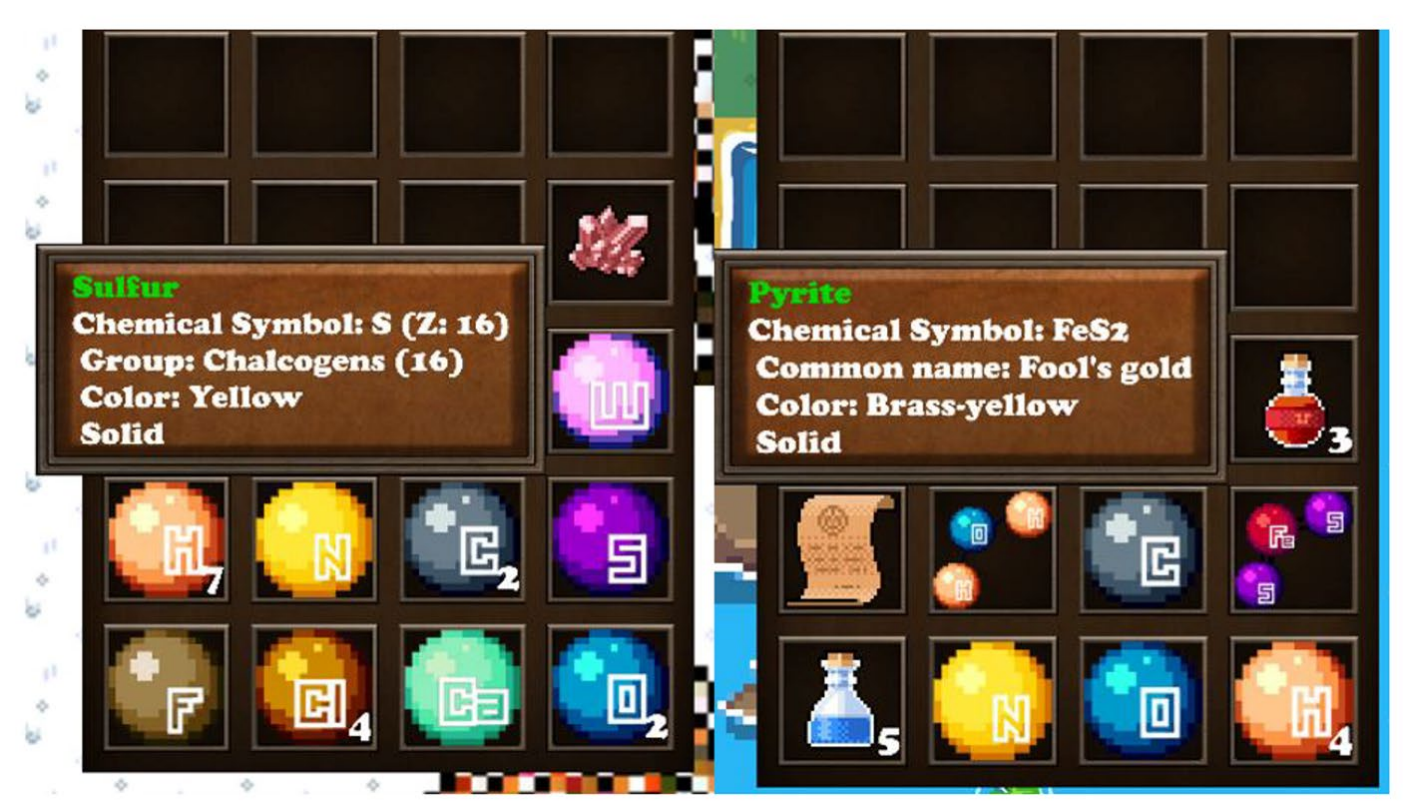
Two instances of the inventory and tooltip implementation

One of the main components of the gameplay is the inventory (Fig. 3). The character stores the items or elements that are collected through the map in the inventory (representation: game world representation) and more information can be seen in the form of a tooltip, by hovering the mouse cursor over them. The information for elements further abets the educational role of the game, as it includes the chemical symbol and atomic number, the periodic table group name and number, its color and the phase in standard temperature and pressure (STP) (context: support, pedagogy: associative, situative). For compounds and minerals, information also includes the common name. The player can re-organize the items in the inventory by clicking on them and dropping it. Depending on the drop position, an item can change slot, return to its original place, two items can swap slots or an item can be added to a stack of similar type items (representation: interactivity).

**Fig. 4:**
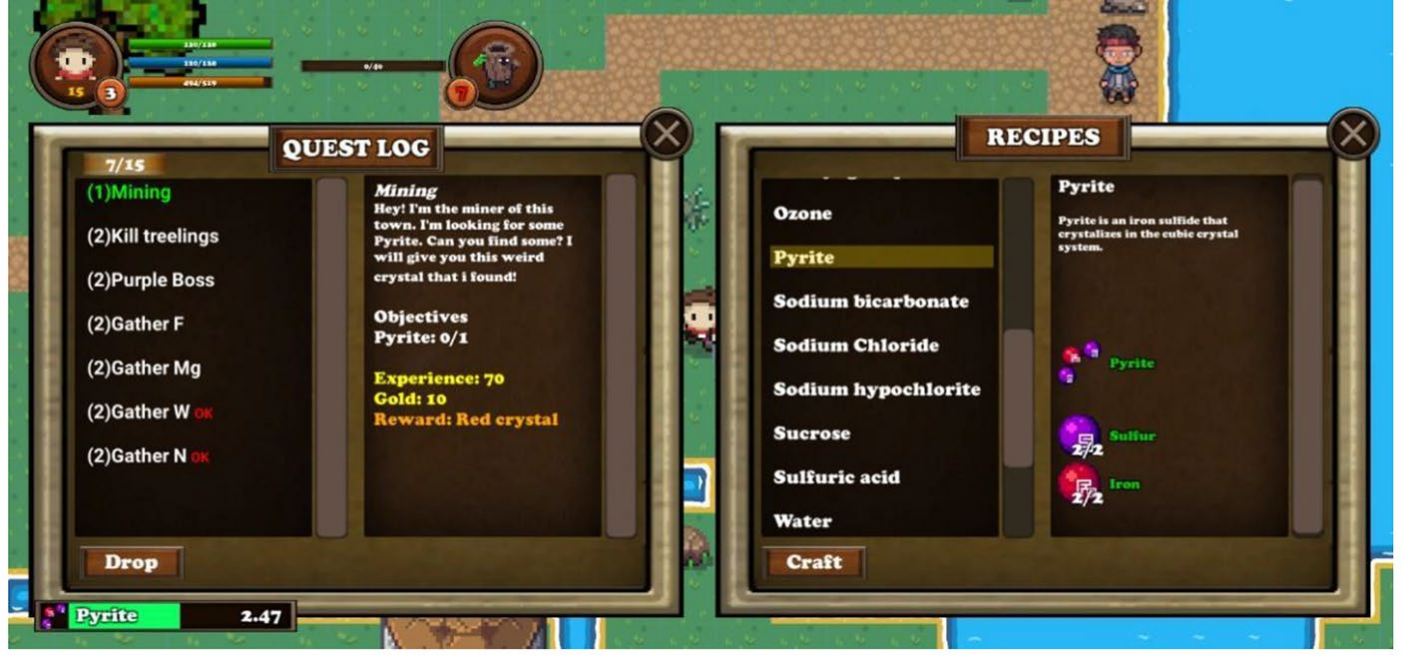
Quest and Crafting systems along with the progress bar

During the game, the player has to complete a number of quests (pedagogy: cognitive, associative, situative). Those quests are of two types: collection quests (including gathering or crafting quests) or elimination quests. The player can refer to the quest window (shown in Fig. 4), at any time during game playing, in order to get information for active quests (context: support). By clicking on a quest, its title turns green, indicating that it is selected and information appears regarding the quest’s demands and the completion rewards. Completed quests are accompanied by a red colored ‘OK’ and remain in the quest window till the player returns to the original NPC. NPCs that hold quests are marked with a yellow exclamation mark, which changes to a gray question mark after the player has accepted the quest or a yellow question mark after the quest objective is completed and the character has to return and claim his rewards (pedagogy: associative).

The crafting system is a game mechanic of major importance both for gameplay and for the educational aspect of Elementium (pedagogy: associative, situative, cognitive). The player can view the crafting objective of a quest from the quest window and find the recipe at the recipes window. By clicking on a recipe title, information is displayed, along with the components required for the craft (context: support). If the player has the number of components required in his inventory, the crafting can be initiated by pressing the corresponding button. When the button is pressed, the progress bar pops up, in the same manner as with the casting of a spell, previously described. After the countdown, the newly crafted item can be found in the inventory, while the required constituents are consumed.

**Fig. 5:**
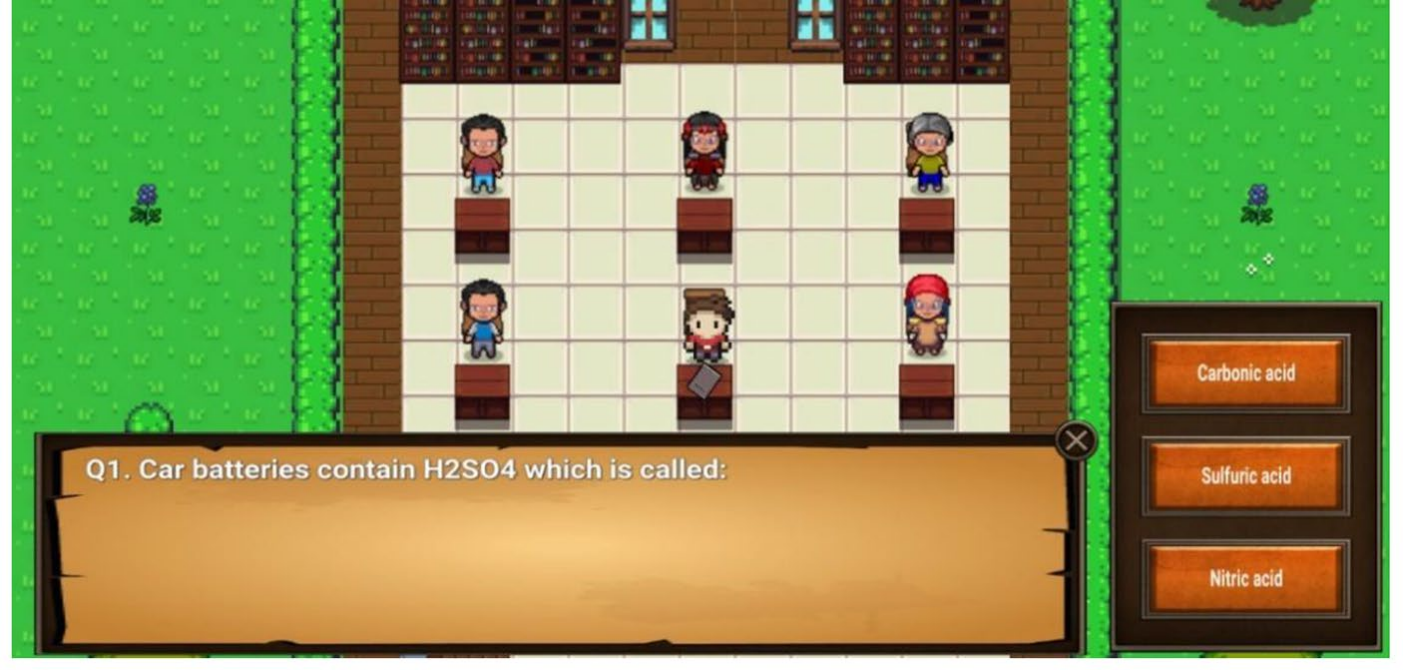
Question system implementation

Another important component of Elementium is the questions system presented in Fig. 5. At the end of each area the player is presented with a set of Chemistry based multiple choice questions (pedagogy: cognitive). The questions are related to the Chemistry topics that the player faced in the previous area or are general questions, directly taken from the Chemistry textbooks. If the player chooses the wrong answer, a message appears indicating the mistake. At the end of a failed attempt, the number of correct answers is displayed and the set of questions has to be replayed. When all answers are answered correctly, the player gets a special key as a reward. Altogether, through the game, 70 different questions must be correctly answered (pedagogy: associative, representation: interactivity).

**Fig. 6:**
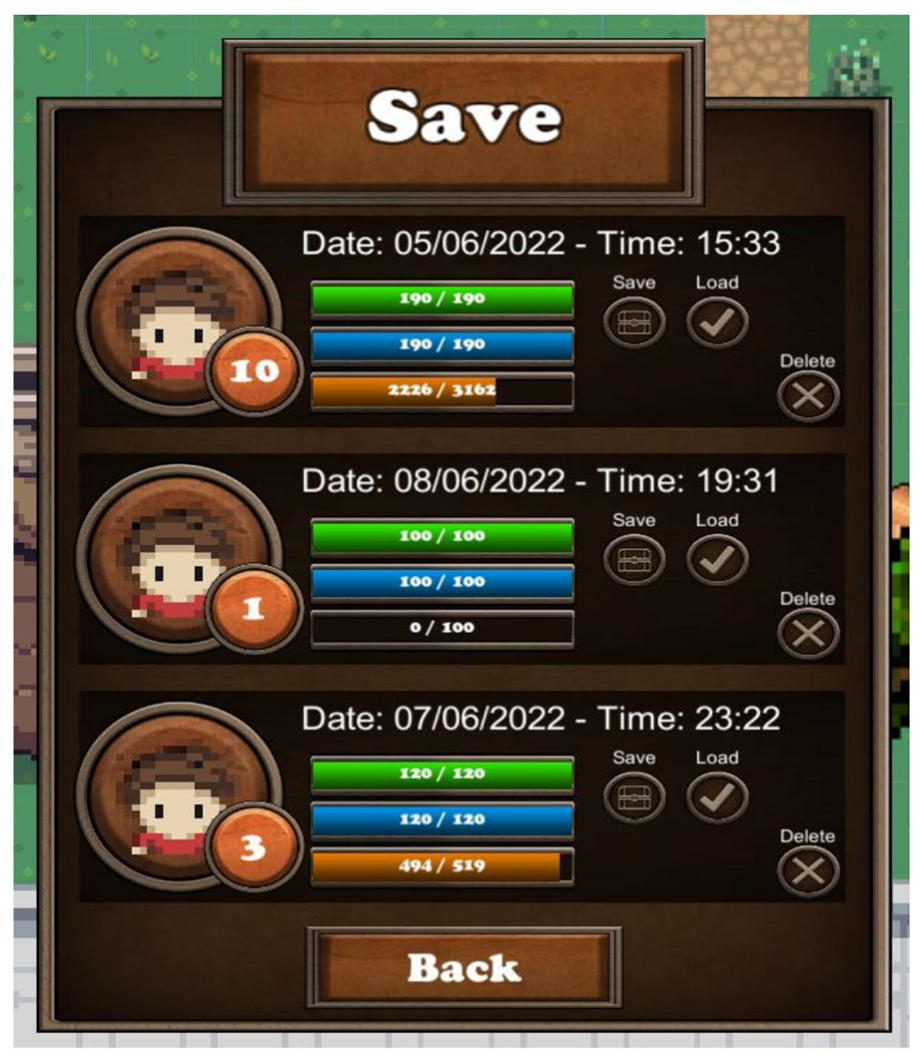
The save menu

Lastly, the saving system is responsible for saving and loading the game (context: place). The save menu, accessed via the game menu, contains three slots that are initially empty. If a player clicks the save button next to a slot, a game instance is saved and the slot is occupied with the main characters stats, like health, mana and experience points, gold, active quests etc. (Fig. 6).

## Methods

### Instruments

For an early evaluation of the game, teachers were invited to play-test the game at leisure in their homes and evaluate it through an anonymous questionnaire. The questionnaire included:

- demographic questions.
- questions regarding the proper execution of the game.
- questions based on the acceptability, usability and didactic utility dimensions of key criteria of the game design model proposed by Sanchez (2011).
- questions on the game environment proposed in other game design and evaluation frameworks (Bellotti et al., 2011; De Gloria et al., 2014; de Freitas & Jarvis, 2006; Ibrahim & Jaafar, 2009; Malliarakis et al., 2014; Petri et al., 2018) and utilized in a similar evaluation of the game MYTH TROUBLES (Evangelopoulou & Xinogalos, 2018).
- two open-ended questions on the interesting features of the game and proposals for improvements.

The key criteria for game design by Sanchez have been extensively utilized for evaluating educational serious games during their design (Marfisi-Schottman et al., 2014), as well as quality indicators during the evaluation of educational SGs (Abarkan & BenYakhlef, 2022; Evangelopoulou & Xinogalos, 2018). Moreover, the criteria used for evaluating the game environment are proposed in several SG design and evaluation frameworks (Bellotti et al., 2011; De Gloria et al., 2014; de Freitas & Jarvis, 2006; Ibrahim & Jaafar, 2009; Malliarakis et al., 2014; Petri et al., 2018) and consequently the questionnaire was considered to be validated in existing literature.

Following, we elaborate on the quality indicators that were investigated with the aforementioned questionnaire:

- *Acceptability*: this dimension includes questions for evaluating the impact of the game in the learning process and consequently the acceptance of the game as a learning aid by teachers, students and the institution (Sanchez, 2011). Four questions were included in the questionnaire for evaluating the content of the game in terms of its relevance, fitness both for the curriculum and students’ profile, and finally its suitability for use in the class in terms of the time needed to play it.
- *Usability*: this dimension includes questions for evaluating various technical and pedagogical factors and deciding whether the game can be used in a specific learning context/process (Sanchez, 2011). Six questions were included in the questionnaire for evaluating whether the game can run on school/student devices, has a reasonable learning (to play) curve, provides guidance and adequate help, provides clear and relevant feedback, and finally whether it can be used at school and/or for self-studying.
- *Didactic Utility*: this dimension includes questions for evaluating the didactical utility, or else the pedagogical features, of the game (Sanchez, 2011). Four questions were used for evaluating whether the game is suited to the pedagogical objectives of the teacher and its tasks are relevant for achieving the intended learning goals, as well as the degree that students are expected to improve their knowledge and develop relevant competencies through playing.
- *Game environment*: this dimension includes questions on the attractiveness (Petri et al., 2018; Sanchez, 2011) and the user-friendliness of the game environment (Petri et al., 2018; Sanchez, 2011), the scenario (de Freitas & Jarvis, 2006; Malliarakis et al., 2014; Petri et al., 2018), the degree that in-game components keep students’ interest undiminished (Petri et al., 2018), the opportunity for self-assessment (Ibrahim & Jaafar, 2009), and finally the balance between game components and the achievement of the educational objective (Bellotti et al., 2011; De Gloria et al., 2014).

A five-point Likert scale was used for all the questions (with the exception of the two open-ended questions), where 1 equals ‘strongly disagree’ and 5 equals ‘strongly agree’.

The questionnaire was created using Google forms and was linked through the game’s webpage (https://alexpheeuom.github.io/elementium/Elementium.html), while it remained open to submissions for one month. The first author made an announcement about the game, its availability and an invitation for its anonymous evaluation by Physics and Chemistry teachers through his social media.

### Sample

The game was played and evaluated anonymously by 26 people, most of them in the age groups 18–28 (38.5%) and 29–39 (57.7%). This research has been conducted following the ethical requirements established by Greece national board of ethics. All the participants hold a Bachelor degree in Physics (76.9%) or Chemistry (23.1%) and have experience in teaching Chemistry. Specifically, the majority of the participants (69.3%) have 3 to 10 years of experience in teaching Chemistry. As for the game execution, all of the participants finished the game, which ran on their PCs with no problems. From the whole of the participants, 7.7% finished the game in approximately 1 h, 53.8% in approximately 2 h and 38.5% needed more than two hours for completing the game.

### Statistical analysis

Data was collected through an anonymous questionnaire (Google form) and was statistically analyzed using MS Excel. We calculated the mean value (x̄), standard deviation (σ) and median (x͂) for each question.

## Results

### Acceptance of the game

Regarding the dimension of *acceptance* of the game (Table 2), the evaluation showed that 92.31% of the participants strongly consider the content relevant and with no errors (Q1, x̄ = 4.92, σ = 0.27, x͂ = 5), while it fits the characteristics of the students (Q2, x̄ = 4.81, σ = 0.40, x͂ = 5). Furthermore, 96.15% of the participants strongly agree that the content is relevant with the junior high school Chemistry textbook, which is actually true since the majority of the educational content derived from the textbook (Q3, x̄ = 4.96, σ = 0.20, x͂ = 5). For the last question of this set, participants were asked whether the time required for the gameplay allows for its use during a class of Chemistry and the results are quite diversified. Whereas there are a few answers that agree with this sentence, there is also a 15.38% of neutral answers and a major 76.93% that disagrees (or strongly disagrees), meaning that Elementium, at its current form, is not suitable for use in the classroom (Q4, x̄ = 2.00, σ = 1.02, x͂ = 2).

**Table 2:** Results on game acceptance

Question	Code	Mean Value	Median	Std. deviation	Strongly disagree	Disagree	Neutral	Agree	Strongly agree
The content is relevant (no errors)	Q1	4.92	5	0.27	0 (0%)	0 (0%)	0 (0%)	2 (7.69%)	24 (92.31%)
The content fits the characteristics of the students (age, prior knowledge etc.)	Q2	4.81	5	0.40	0 (0%)	0 (0%)	0 (0%)	5 (19.23%)	21 (80.77%)
The content fits the curriculum of junior high school Chemistry textbook	Q3	4.96	5	0.20	0 (0%)	0 (0%)	0 (0%)	1 (3.85%)	25 (96.15%)
The time devoted to play the game allows its use in class	Q4	2.00	2	1.02	9 (34.62%)	11 (42.31%)	4 (15.38%)	1 (3.85%)	1 (3.85%)

### Usability of the game

Table 3 shows the results for the dimension of usability. Almost all of the participants (96.15%) strongly agree that Elementium will have no problem running in school or students’ personal computers, something that probably rises from the fact that none of the participants faced problems during installation or execution (Q5, x̄ = 4.96, σ = 0.20, x͂ = 5). A large percentage of the respondents (42.31%) agree that the time that is required to learn how to use the game is reasonable, while 57.69% strongly agree with the same statement (Q6, x̄ = 4.58, σ = 0.50, x͂ = 5). Moreover, 76.92% of the participants agree that the game provides sufficient guidance and adequate help (Q7, x̄ = 4.15, σ = 0.46, x͂ = 4), whilst 53.85% agree and 46.15% strongly agree that Elementium provides clear and relevant feedback to the student (Q8, x̄ = 4.46, σ = 0.51, x͂ = 4). The positive results to the last two questions probably emerge from implemented game components such as the immediate feedback after an answer to the NPCs’ questions, the existence of NPCs that provide help and tips, and finally the tooltips and the help window, available anytime during gameplay. The responses to the question ‘The game can be used at school’, are diversified, much like the corresponding question (Q4) of the previous set. In particular, the answers to this question display the greater value of standard deviation, since 11.54% of the participants agree or strongly agree, 30.77% are neutral and 57.69% disagree or strongly disagree with the statement (Q9, x̄ = 2.42, σ = 1.10, x͂ = 2). The majority of the answers are in the negative spectrum, strengthening the opinion that the game is not meant for playing during a Chemistry class. On the other hand, 34.62% of the respondents agree and 65.38% strongly agree that Elementium can certainly be used as a supplementary tool to aid the user while studying at home (Q10, x̄ = 4.65, σ = 0.49, x͂ = 5).

**Table 3:** Results on the usability of the game

Question	Code	Mean Value	Median	Std. deviation	Strongly disagree	Disagree	Neutral	Agree	Strongly agree
The game can run on school devices (or students’ personal devices)	Q5	4.96	5	0.20	0 (0%)	0 (0%)	0 (0%)	1 (3.85%)	25 (96.15%)
The time devoted to learning how to use the game is reasonable	Q6	4.58	5	0.50	0 (0%)	0 (0%)	0 (0%)	11 (42.31%)	15 (57.69%)
The game provides guidance and adequate help	Q7	4.15	4	0.46	0 (0%)	0 (0%)	1 (3.85%)	20 (76.92%)	5 (19.23%)
The game provides clear and relevant feedback	Q8	4.46	4	0.51	0 (0%)	0 (0%)	0 (0%)	14 (53.85%)	12 (46.15%)
The game can be used at school	Q9	2.42	2	1.10	5 (19.23%)	10 (38.46%)	8 (30.77%)	1 (3.85%)	2 (7.69%)
The game can be used for supporting students in self-studying	Q10	4.65	5	0.49	0 (0%)	0 (0%)	0 (0%)	9 (34.62%)	17 (65.38%)

### Didactic utility of the game

The results for the *didactic utility* of the game are presented in Table 4. As can be seen in Table 4, 80.77% of the educators that participated in the evaluation, agree that the game aids the pedagogical objectives of the teacher (Q11, x̄ = 4.12, σ = 0.43, x͂ = 4), while the vast majority (92.31%) strongly agree that the tasks that the students complete within the game are relevant to the curriculum (Q12, x̄ = 4.92, σ = 0.27, x͂ = 5). It is of major significance that the preponderance of the respondents (93.15%) strongly agrees that through their occupation with the game, students are expected to improve their knowledge (Q13, x̄ = 4.96, σ = 0.20, x͂ = 5). Finally, for the last question of this set, most of the participants agree or strongly agree that through the gameplay, students will develop relevant competencies (Q14, x̄ = 4.15, σ = 0.54, x͂ = 4).

**Table 4:** Results on the didactic utility of the game

Question	Code	Mean Value	Median	Std. deviation	Strongly disagree	Disagree	Neutral	Agree	Strongly agree
The game is suited to the pedagogical objectives of the teacher	Q11	4.12	4	0.43	0 (0%)	0 (0%)	1 (3.85%)	21 (80.77%)	4 (15.38%)
The tasks of the students within the game are relevant	Q12	4.92	5	0.27	0 (0%)	0 (0%)	0 (0%)	2 (7.69%)	24 (92.31%)
By playing, students improve their knowledge	Q13	4.96	5	0.20	0 (0%)	0 (0%)	0 (0%)	1 (3.85%)	25 (96.15%)
By playing, students develop relevant competencies	Q14	4.15	4	0.54	0 (0%)	0 (0%)	2 (7.69%)	18 (69.23%)	6 (23.08%)

### Game environment

The results regarding the *game environment* and a general question on the use of educational games in High school can be seen in Table 5. A great percentage of the educators that participated believe that the environment of Elementium is attractive to the student (Q15, x̄ = 4.77, σ = 0.43, x͂ = 5) and in the same time the interface is student friendly (Q16, x̄ = 4.92, σ = 0.27, x͂ = 5). Additionally, 80.77% of the participants strongly agree that the game scenario will raise students’ attention (Q17, x̄ = 4.81, σ = 0.40, x͂ = 5), whereas 88.46% strongly agree that the game components will keep the students’ interest undiminished (Q18, x̄ = 4.88, σ = 0.33, x͂ = 5). The response to these four questions was the desired one, since they all regard major components when it comes to user engagement. Furthermore, 69.23% of the participants agree that the game is expected to motivate the students into studying and self-assessment (Q19, x̄ = 4.31, σ = 0.47, x͂ = 4). The final question of this set contains the most important question of this questionnaire and it regards the balance between game components and the achievement of the educational objective (Q20, x̄ = 4.96, σ = 0.2, x͂ = 5). This was our main goal during the design and development process of Elementium and 25 out of the 26 participants (96.15%) strongly agree that this goal is achieved.

**Table 5:** Results on the game environment

Question	Code	Mean Value	Median	Std. deviation	Strongly disagree	Disagree	Neutral	Agree	Strongly agree
Environment									
The game environment is attractive to students	Q15	4.77	5	0.43	0 (0%)	0 (0%)	0 (0%)	6 (23.08%)	20 (76.92%)
The game environment is student friendly	Q16	4.92	5	0.27	0 (0%)	0 (0%)	0 (0%)	2 (7.69%)	24 (92.31%)
The scenario of the game is interesting for the student	Q17	4.81	5	0.40	0 (0%)	0 (0%)	0 (0%)	5 (19.23%)	21 (80.77%)
In-game components (gathering elements, crafting compounds etc.) keeps the student’s interest undiminished	Q18	4.88	5	0.33	0 (0%)	0 (0%)	0 (0%)	3 (11.54%)	23 (88.46%)
The game environment provides the students an opportunity for self-assessment	Q19	4.31	4	0.47	0 (0%)	0 (0%)	0 (0%)	18 (69.23%)	8 (30.77%)
There is balance between game components and the achievement of the educational objective	Q20	4.96	5	0.2	0 (0%)	0 (0%)	0 (0%)	1 (3.85%)	25 (96.15%)
General									
Educational games like Elementium can be used as supportive material in junior high school classes	Q21	4.31	4	0.55	0 (0%)	0 (0%)	1 (3.85%)	16 (61.54%)	9 (34.62%)

### Interesting features and proposals for improvements

Table 6 shows the results of the two open-ended questions (Q22, Q23) included in the questionnaire. Although only 3 out of the 26 participants filled out these non-compulsory questions, the overall response was positive and the improvements proposed will be taken into account regarding the game’s future progress.

**Table 6:** Answers to the open-ended questions (literal translation from Greek)

Q22. Mention 2–3 game characteristics that you consider interesting	Q23. Suggest your proposals regarding the game’s future improvements
*“Interesting idea and nice music.”*	*“1. Sometimes the character gets stuck behind obstacles* *2. Could contain more detailed instructions* *3. In future updates, a wider range of the Chemistry book syllabus can be added, or maybe more complex concepts.”*
*“The game has nice aesthetic and background music. Interesting idea. Very nice try.”*	*“In the future some gameplay problems must be fixed and then the content can be expanded. Significantly: more elements, subatomic particles, chemical bonds etc.”*
*“Nice aesthetic, reminds of old Gameboy games. Also there is pleasant music and an interesting script.”*	*“The gameplay duration is quite long to use during a class. Perhaps, with the syllabus divided in chapters, the game can be utilized as a supplement for the class.”*

## Discussion

**Fig. 7:**
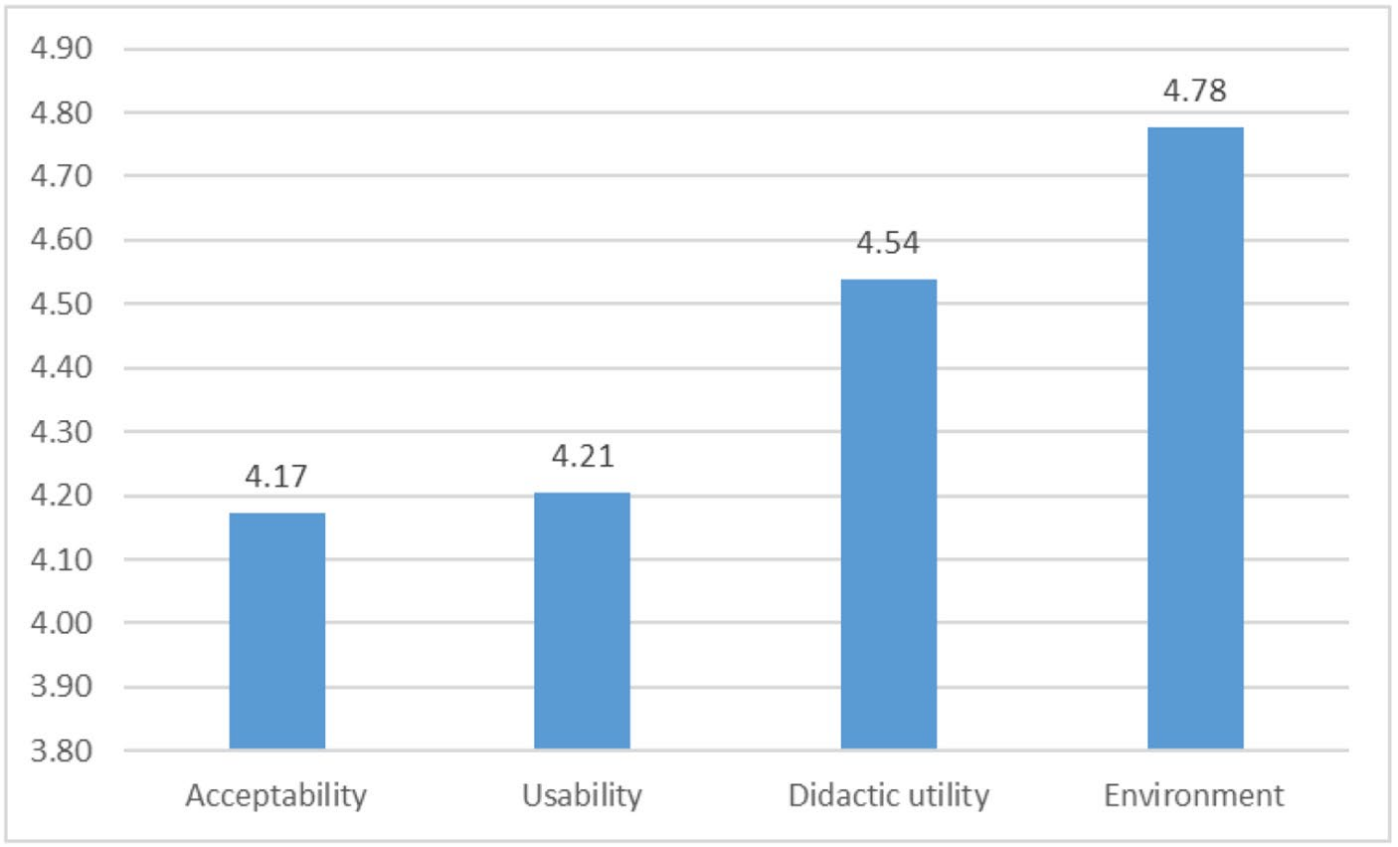
Mean values for each evaluation dimension

Breaking down the questionnaire answers, it is easy to realize that Elementium is well accepted by the educators who play-tested it. The mean value of the answers’ scores for each dimension of criteria investigated through the questionnaire ranges from 4.15 to 4.96 / 5.00 (Fig. 7). The mean value for each category, shown in Fig. 7, is considerably high, with the Environment scoring an impressive 4.78/5.00 and the Didactic utility 4.54/5.00.

As expected, the most negative answers are noted for the two questions (Q4, Q9) regarding the possibility of using the game in school classes, affecting the mean value for the acceptability (Fig. 7: 4.17) and the usability (Fig. 7: 4.21) dimensions. Even though the deviation is high (1.10 and 1.02), the mean value is below 2.5 / 5.00 for each one of these questions, while the median is just 2, something that strengthens the negative opinion. Two major points shape the answers to those questions, as highlighted by the third respondent (Table 6) in the open ended questions, namely the long gameplay time and the absence of chapter separated contents. We must note that the game was initially designed to be used by students as a learning aid for self-learning at home (Ibrahim & Jaafar, 2009) and consequently the aforementioned results were not surprising. However, as noted, in a future version of Elementium more emphasis should be given on learning content modeling, providing more chances for scaffolding (Ibrahim & Jaafar, 2009; Malliarakis et al., 2014). This could be accomplished by splitting the game story into chapters in order for the user to be able to choose from a dedicated menu and make it possible to use the game in the class (Sanchez, 2011) during a didactical hour (45 min). Moreover, the content of the game could be extended, including more elements, chemical bonds or subatomic particles, as proposed by the first two respondents in the open-ended questions (Table 6).

One of the most important results of the study refers to the balance between game components and the achievement of the educational objective (Bellotti et al., 2011; De Gloria et al., 2014) that is considered to be one of the most challenging open issues in SG design (Natucci & Borges, 2021; Silva, 2020). The decision to use an RPG game genre (Baptista 2015; Cheng et al., 2015; Grande-de-Prado, 2020), as well as to interleave the educational content/activities with the quests assigned to players (collecting and crafting elements) and typical game features (such as an inventory for collecting elements) seems to have had a positive impact on achieving the required balance between game and pedagogy (Q20: median = 5). Consequently, RPG games that are considered to be successful for science education SGs (Baptista 2015; Cheng et al., 2015; Grande-de-Prado, 2020) seem to be a good choice for Chemistry games in specific, but further research is necessary in order to confirm this.

## Limitations of the study

Elementium is a serious game for Chemistry targeted at high school students. As is the case with any game-based learning approach, three dimensions have to be taken into account in order to ensure its effective application in an educational context (Sanchez, 2011). These dimensions include the game’s acceptability by students, teachers and the institution, its usability and didactic utility (Sanchez, 2011). As Evangelopoulou and Xinogalos (2018; p. 90) state *“the acceptance of the game by teachers and their intention to utilize it in the classroom is considered important taking into account the scepticism of instructors to incorporate games in their courses”*. In the case that a teacher evaluates positively the added value of a game in the learning process, then the game can be utilized in the classroom or be proposed to students as a learning aid that can be used for studying at home (Ibrahim & Jaafar, 2009).

In this sense, the pilot evaluation of Elementium was carried out by teachers that have experience in teaching Chemistry. Since the participants are experts in the area of the game, it is considered that the results of the evaluation are trustworthy. Specifically, as noted by Nielsen and Molich (1990) two to three experts in the area of the information system under evaluation and usability issues can bring to surface nearly 81–90% of usability problems.

It is clear that both the approach and the sample of the study pose limitations on the conclusions drawn. Taking into account the positive evaluation of the game by the teachers, the next step in the process is the evaluation of the game by students or anyone interested in learning Chemistry in order to draw safe conclusions about its didactical effectiveness. This could be accomplished through a study carried out with junior high school students in a formal educational setting. Specifically, an experimental group of students could utilize Elementium in the classroom or at home and a control group could be taught without it, while a pretest and posttest design could be utilized for investigating potential changes that could be attributed to the use of game.

## Conclusions

This article presents the results of a pilot study that followed the design and implementation of a SG for familiarizing junior high school students with the basics of Chemistry. Several SGs for Chemistry have been developed, but most of them fall short in the essential fun factor that characterizes a SG. Our goal was to incorporate the educational content into the mechanics of a video game and deliver an entertaining game that will be attractive to students. Elementium’s design was guided by the Four-Dimensional framework (de Freitas & Jarvis, 2006) and its pilot evaluation was based on the questionnaire proposed by Sanchez (2011) and expanded taking into account relevant literature (Bellotti et al., 2011; De Gloria et al., 2014; de Freitas & Jarvis, 2006; Evangelopoulou & Xinogalos, 2018; Ibrahim & Jaafar, 2009; Malliarakis et al., 2014; Petri et al., 2018). Elementium was positively evaluated in terms of its acceptance, usability, didactic utility and game environment by Chemistry teachers. The game was considered to be successful in achieving a good balance between game and learning and this was heavily attributed to the decision to design it as an RPG game (Baptista 2015; Cheng et al., 2015; Grande-de-Prado, 2020) and interleave game features with educational material/tasks through its scenario. The pilot evaluation showed that Elementium can form a sufficient learning environment and be utilized as supportive material to the teacher’s educational goals. Specifically, the game was considered to be appropriate for self-studying at home and with specific modifications (separating the educational material in chapters, or else independent game levels) for usage in class.

## Data Availability

The dataset analysed during the current study is available from the corresponding author on reasonable request.
